# Phase I/II clinical trial of efficacy and safety of EGCG oxygen nebulization inhalation in the treatment of COVID-19 pneumonia patients with cancer

**DOI:** 10.1186/s12885-024-12228-3

**Published:** 2024-04-17

**Authors:** Xiaoyan Yin, Wanqi Zhu, Xiaoyong Tang, Guangjian Yang, Xianguang Zhao, Kaikai Zhao, Liyang Jiang, Xiaolin Li, Hong Zhao, Xin Wang, Yuanyuan Yan, Ligang Xing, Jinming Yu, Xiangjiao Meng, Hanxi Zhao

**Affiliations:** 1 Department of Radiation Oncology, Shandong Cancer Hospital and Institute, Shandong First Medical University, Shandong Academy of Medical Sciences, Jiyan Road 440, 250117 Jinan, Shandong China; 2 Department of Medical Oncology, Shandong Cancer Hospital and Institute, Shandong First Medical University, Shandong Academy of Medical Science, 250117 Jinan, Shandong Province China; 3 Department of Radiology, Shandong Cancer Hospital and Institute, Shandong First Medical University, Shandong Academy of Medical Science, 250117 Jinan, Shandong Province China

**Keywords:** COVID-19 pneumonia, Epigallocatechin-3-gallate, Cancer, Safety, Efficacy

## Abstract

**Background:**

The antiviral drug Nirmatrelvir was found to be a key drug in controlling the progression of pneumonia during the infectious phase of COVID-19. However, there are very few options for effective treatment for cancer patients who have viral pneumonia. Glucocorticoids is one of the effective means to control pneumonia, but there are many adverse events. EGCG is a natural low toxic compound with anti-inflammatory function. Thus, this study was designed to investigate the safety and efficacy of epigallocatechin-3-gallate (EGCG) aerosol to control COVID-19 pneumonia in cancer populations.

**Methods:**

The study was designed as a prospective, single-arm, open-label phase I/II trial at Shandong Cancer Hospital and Institute, between January 5, 2023 to March 31,2023 with viral pneumonia on radiographic signs after confirmed novel coronavirus infection. These patients were treated with EGCG nebulization 10 ml three times daily for at least seven days. EGCG concentrations were increased from 1760-8817umol/L to 4 levels with dose escalation following a standard Phase I design of 3–6 patients per level. Any grade adverse event caused by EGCG was considered a dose-limiting toxicity (DLT). The maximum tolerated dose (MTD) is defined as the highest dose with less than one-third of patients experiencing dose limiting toxicity (DLT) due to EGCG. The primary end points were the toxicity of EGCG and CT findings, and the former was graded by Common Terminology Criteria for Adverse Events (CTCAE) v. 5.0. The secondary end point was the laboratory parameters before and after treatment.

**Result:**

A total of 60 patients with high risk factors for severe COVID-19 pneumonia (factors such as old age, smoking and combined complications)were included in this phase I-II study. The 54 patients in the final analysis were pathologically confirmed to have tumor burden and completed the whole course of treatment. A patient with bucking at a level of 1760 umol/L and no acute toxicity associated with EGCG has been reported at the second or third dose gradients. At dose escalation to 8817umol/L, Grade 1 adverse events of nausea and stomach discomfort occurred in two patients, which resolved spontaneously within 1 hour. After one week of treatment, CT showed that the incidence of non-progression of pneumonia was 82% (32/39), and the improvement rate of pneumonia was 56.4% (22/39). There was no significant difference in inflammation-related laboratory parameters (white blood cell count, lymphocyte count, IL-6, ferritin, C-reactive protein and lactate dehydrogenase) before and after treatment.

**Conclusion:**

Aerosol inhalation of EGCG is well tolerated, and preliminary investigation in cancer population suggests that EGCG may be effective in COVID-19-induced pneumonia, which can promote the improvement of patients with moderate pneumonia or prevent them from developing into severe pneumonia.

**Trial registration:**

ClinicalTrials.gov Identifier: NCT05758571. Date of registration: 8 February 2023.

**Supplementary Information:**

The online version contains supplementary material available at 10.1186/s12885-024-12228-3.

## Introduction

SARS-Cov-2 is a type of novel coronavirus that has caused severe acute respiratory syndrome spread around the world since the end of 2019 [1, 2] Patients with characteristics as older age, smoking history or complications such as cardiovascular disease, diabetes, obesity, and cancer are at high risk for severe COVID-19 and related severe events [1, 3–5]. It has been described that compared with patients without those condition, patients with prespecified coexisting disease were about approximately twice as likely to exacerbate to severe COVID-19 and five-fold high mortality rate [6, 7]. Undoubtably, cancer patients were more vulnerable to SARS-CoV-2 and show more deteriorating conditions and poor outcomes than individuals without cancer because of their systemic immunosuppressive state caused by the malignancy and anticancer treatments [8–10]. Most importantly, patients with cancer were observed to be older, more likely to have a history of smoking and more severe baseline CT manifestation [9], which possess more risk factors for severe COVID-19.

SARS-Cov-2 induces the uncontrolled release of excessive inflammatory cytokines by host immune response [11], resulting in mild to severe pneumonia, acute lung injury and even hypoxic respiratory failure. This over-production of pro-inflammatory cytokines induces more damage to the host cells than the one induced by SARS-CoV-2 as pathogen invader [12]. Although Nirmatrelvir or other specific therapeutics could reduce viral load and the incidence of severe COVID-19 in the early stage of viral infection, the inhibition of inflammatory response, elimination of pulmonary inflammation and repair of injury in patients with COVID-19 are also the important consideration in clinic [13]. However, for hyper-inflammation, there are currently no effective and low side-effect drugs to curb the development of pneumonia except corticosteroids or cytokine-directed biological agents.

Epigallocatechin-3-gallate (EGCG), the major and most highly bioactive constituent in green tea, has strong anti-inflammatory, anti-oxidant, antiviral and even antitumor activities [14, 15]. Some studies found EGCG can downregulate the expression of inflammatory mediators and signal transduction pathways by acting on signal transducers and activators of transcription (STAT) 1/3 and nuclear factor kappa-light-chain-enhancer of activated B cells (NF-κB) transcription factors [12], which could be considered as a potential nature immune homeostasis agent to counteract hyper-inflammation existing in COVID-19.

In our previous studies [16–19], we found that EGCG can prevent and cure radiation-induced normal tissue damage in tumor patients with high safety and potent anti-inflammatory competence. Other clinical studies also documented favorable security of EGCG. Based on these encouraging results, we conducted this phase I-II clinical trial to investigate the possible role of EGCG aerosol inhalation in the treatment of interstitial pneumonia in tumor patients infected with novel coronavirus.

## Methods

### Participants

Recruitment into this trial was during January 5, 2023 to March 31,2023. The total of 60 patients were recruited at Shandong Cancer Hospital and Institute in Shandong, China. Eligibility criteria included patients 18 years or older who had histologically diagnosed malignant tumors; being or has been confirmed SARS-CoV-2 infection; with moderate pneumonia, according to the definition of COVID-19 Infection Diagnosis and Treatment Plan (version 10) issued by the National Health Commission of the PRC (in Supplement file); the pulmonary function of the patient can be treated with aerosol inhalation for 7 days. Exclusion criteria included current or recent progresses rapidly and may develop into a critical illness with coronavirus in a short period of time; non-SARS-CoV-2 infectious interstitial pneumonia; using immune suppressive agents during administration. Low doses of dexamethasone (mostly less than 5 mg/d or equivalent doses of other kinds of glucocorticoids) were also allowed for some patients with brain metastases, which are inevitable. The study was approved by the Institutional Review and Ethical Committees at the affiliated Cancer Hospital of Shandong first Medical University and registered at ClinicalTrials.gov (NCT05758571) (02/08/2023). Informed consent was provided by every participant. This study followed the Consolidated Standards of Reporting Trials (CONSORT) reporting guideline.

### Study design and treatment

EGCG (high pressure liquid chromatographic purity ≥ 98% from Ningbo Hepu Biotechnology Co., Ltd.) is dissolved in 0.9% normal saline. We have chosen a dose of 1760 umol/L as the lower limit for this phase I study (by referring to previous studies). Four dose levels for EGCG were defined as following: 1760, 3520, 5878 and 8817 umol/L per dose. Dose escalation proceeded according to a standard phase I design with three patients initially treated on each tier. If, on any dose tier of EGCG, two of three patients or two of six patients experienced toxicity due to EGCG, dose escalation of EGCG would cease. Before the dose of EGCG was escalated, the whole patients on a tier were required to be observed for a week after starting treatment. The patient inhaled 10 ml EGCG by atomization three times a day. The duration of the study treatment will last for 7 days and the patient may continue to take medication according to their wishes, but the total duration of medication shall not exceed 14 days. The toxicity of EGCG was graded according to NCI CTCAE (National Cancer Institute for Common Terminology Criteria for Adverse Events) v. 5.0. Any grade 1 adverse event caused by EGCG was considered a dose-limiting toxicity. The maximally tolerated dose (MTD) was defined as the highest dose with fewer than one-third of patients experiencing a dose-limiting toxicity (DLT). The recommended dose level of EGCG in Phase II was the highest concentration in the MTD observation. Steroids, non-steroidal anti-inflammatory drugs or other antibiotic/antifungal therapy were not given, unless brain edema caused by brain metastasis or confirmed fungal or bacterial infection. Furthermore, finger pulse oxygen saturation was tested at least once a day to avoid unresponsive patients who progressed to severe cases with silent hypoxia.

### Outcome measures

Safety assessment was based on the NCI Common Terminology Criteria for Adverse Events v. 5.0 to record any adverse events that may be related to EGCG during treatment and within 7 days after treatment. For the evaluation of efficacy, primary outcome was results of the imaging examination (chest enhanced CT scanning) before and after 7 days of EGCG aerosol inhalation therapy. Secondary outcomes were the improvement of general condition and the changes of Inflammation laboratory indexes.

### Radiology evaluation

CT evaluation criteria refer to Chen Yousan’s chest CT score criteria, the common SARS-CoV-2 infectious CT performance: the initial CT evaluation showed multiple small ground glass nodules with patchy lesions distributed along the broncho-vascular bundle; central consolidation with surrounding ground glass density shadows, single or multiple central ground glass nodules or patches, unilateral or bilateral consolidation; multiple patchy, segmental, or large ground glass density shadows or interlobular septal thickening or consolidation in the subpleural or multicenter distribution of both lungs. We classified the treatment outcomes of COVID-19-related pneumonia into three categories. Improvement: the re-evaluation of CT showed that the range of inflammation was limited to the initial CT imaging, and showed consolidation, absorption or fibrosis, or even disappeared completely. Deterioration: the scope of the lesion exceeds the initial CT image or new inflammatory lesions appear. Stable is between improvement or deterioration. Two senior imaging physicians and one clinical physician independently interpret and verify the interpretation opinions on site. If there are any differences in interpretation, they will discuss together until a consensus is reached.

### Safety and inflammation indexes assessments

Safety assessments were performed according to the National Cancer Institute Common Terminology Criteria for Adverse Events, version 5.0. Some adverse events and the changes of Inflammation indexes were monitored using laboratory tests, such as complete blood cell count, chemistry profile, and liver function tests.

### Statistical analysis

The safety assessments were recorded daily from the start of administration to its completion. The efficacy analysis set included patients who completed the composite assessment by the investigators. Statistical analyses were performed using SPSS Statistics for Windows, version 26.0. Measurement data are expressed as mean and SD and analyzed by t test. All the P values are two-sided level of significance of 0.05.

## Result

### Patient characteristics

**Fig. 1:**
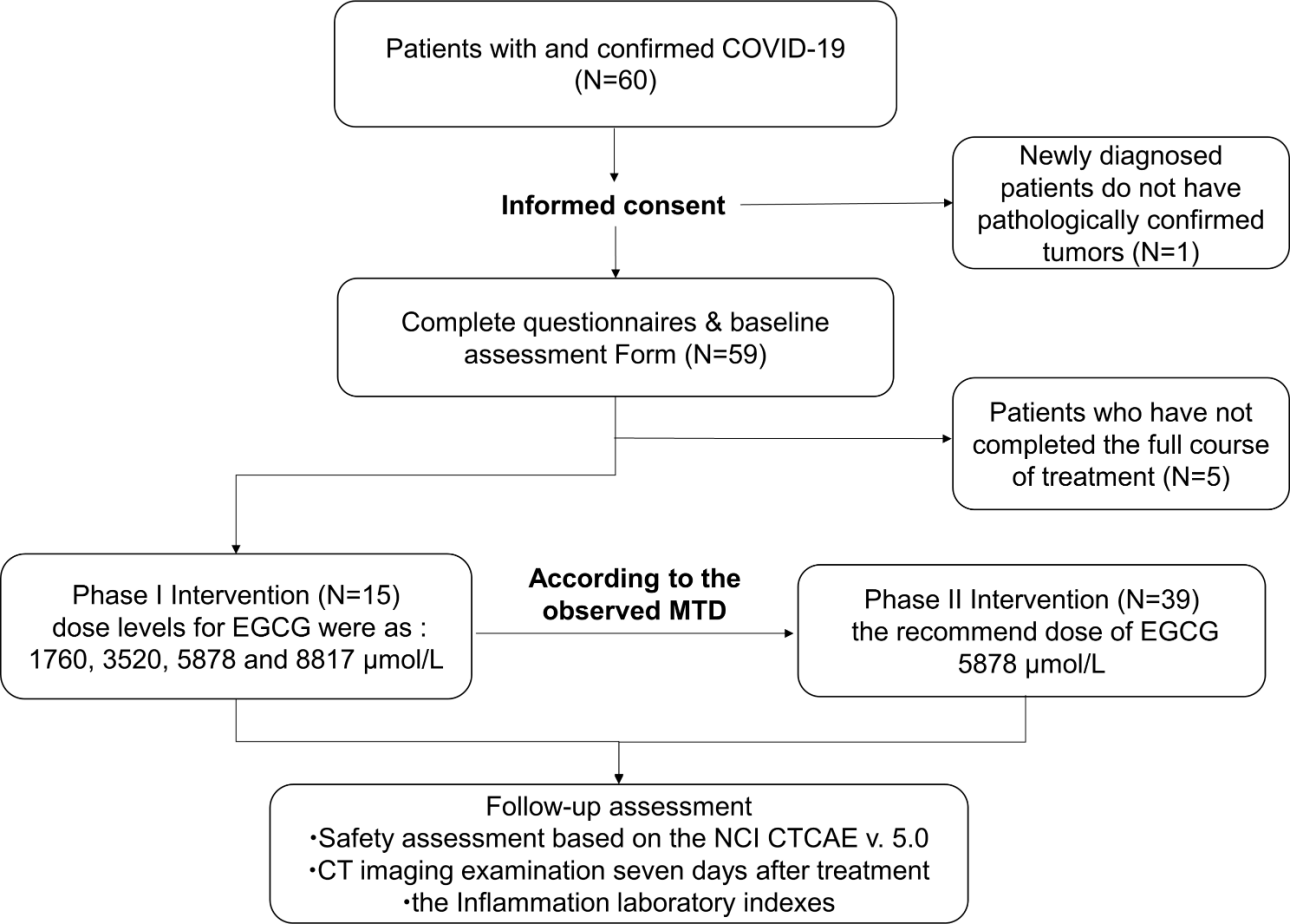
Enrollment and trial flow diagram. EGCG, epigallocatechin-3-gallate; MTD, maximum tolerated dose; NCI CTCAE, National Cancer Institute Common Terminology Criteria for Adverse Events

A total of 60 patients with high risk factors for severe COVID-19-induced pneumonia in the high incidence period of COVID-19 were included from January 5, 2023 to March 31 2023 in this phase I-II trail (Fig. 1). Only one patient did not meet the eligibility criteria (such as no puncture pathological result to determine cancer) and five patients stopped taking drugs on their own and did not complete the whole course of treatment were excluded from the primary analysis. 54 patients completed the course of therapy and were finally analyzed. The patients’ clinical characteristics, baseline demographics and laboratory indictors are listed in Table 1. The median age of patients was 68 years (range from 37 to 78 years old), 44 (81%) were male and the types of cancer include lung cancer (70%), esophageal cancer (24%), pleural mesothelioma (4%), breast cancer (2%). Among the participants, the majority of tumor American Joint Committee on Cancer (AJCC) stage were ≥ III (90%). Regarding the smoking history, 26 patients (48%) were former or current smoker. Eastern Cooperative Oncology Group Performance Status (ECOG PS) of 0–1 and ≥ 2 accounted for 43% and 57% respectively. Additionally, 6 of the 54 patients with brain metastases received dexamethasone combined with mannitol intravenous drip to alleviate the discomfort caused by brain edema. Different doses of dexamethasone were given according to the specific conditions of the patients (2 cases of 2 mg, 1 case of 2.5 mg, 1 case of 3 mg, 1 case of 5 mg and 1 case of 6.5 mg/range from:2 mg to 6.5 mg). Meanwhile, five patients were treated with 40 mg or 60 mg of methylprednisolone in combination with EGCG, considering their large tumor burden, weak physical condition, and accompanying obvious pneumonia symptoms. And there were three patients among them administrated 40 mg methylprednisolone, which is equal to 5 mg dexamethasone for 4 days. A patient received 60 mg for 4 days. The other one received 60 mg for 7 days. 7 patients (13%) received quinolone or aminoglycoside antibiotics and fluconazole antifungal drugs after confirmed bacterial or fungal infection. All patients received standard antiviral treatments in the guideline, of which 18 patients (33%) received Nirmatrelvir/Ritonavir (Paxlovid), 26 patients (48%) received Azvudine, and 10 (19%) received Simnotrelvir/Ritonavir (XIANNUOXIN). Most cancer patients were associated with elderly, who have multiple chronic co-morbidities. 39 patients (72%) had ≥ 3 risk factors. 28 (52%) and 5 (9%) were over 65 years old and obesity (BMI > 30), respectively. 19 of the 54 had long-term use of corticosteroids or recent use of immune checkpoint inhibitors. The particular risk factors for severe and critical COVID-19 high-risk patients are listed in Table 2.

**Table 1:** The patient clinical characteristics, baseline demographics and laboratory indictors

Factor	Patients (%)
	Total *n* = 54
**Gender**	
Male	44(81%)
Female	10(19%)
**Age, median (range, years)**	68 (37–78)
≥ 65	31(57%)
< 65	23(43%)
**Histological subtype**	
Lung cancer	38(70%)
Esophageal cancer	13(24%)
Pleural mesothelioma	2(4%)
Breast cancer	1(2%)
**Smoking history**	
Never smoke	28(52%)
Former/current smoker	26(48%)
**ECOG performance status**	
0–1	23(43%)
≥ 2	31(57%)
**AJCC stage**	
IA	1(2%)
IB	2(4%)
IIA	2(4%)
IIIA	7(13%)
IIIB	16(29%)
IIIC	6(11%)
IV	20(37%)
**Combined with corticosteroid**	
Yes	11(20%)
No	43(80%)
**Combined with antibiotic or antifungal drug**	
Yes	7(13%)
No	47(87%)
**Oxygen saturation, %**	95 (93–97)
**Respiratory rate**	23(17–29)
**Respiratory support**	
No ventilator support*	26(48%)
Non-invasive ventilation	28(52%)
**Laboratory parameters**	
**White blood cell, x 10^9/L**	5.9(3.4–9.6)
**Lymphocyte, x 10^9/L**	1.6(0.1–3.2)
**IL-6, pg/mL**	11.6(2.4–154)
**Ferritin, ng/mL**	305(11.2–1550)
**C-reactive protein, mg/L**	11.7(0.3-104.6)
**Lactate dehydrogenase, u/L**	241(144–1245)

*include patients receiving low- or medium-flow nasal oxygen. ECOG PS, Eastern Cooperative Oncology Group Performance Status; AJCC stage, American Joint Committee on Cancer stage; IL-6, Interleukin-6

**Table 2:** Risk factors of severe/critical COVID-19 (pneumonia) patients

Risk Factors	Patients (%) Total = 54
**Age (> 65 years)**	28(52)
**Obesity (BMI > 30)**	5(9)
**Smoke**	26(48)
**Cardiovascular and cerebrovascular diseases** ^**a**^	23(43)
**Diabetes Mellitus**	11(20)
**Immunodeficiency** ^**b**^	19(35)
**Malignancy**	54(100)
**Chronic lung disease**	7(13)
**Chronic kidney disease**	1(2)

(a) include patients with hypertension (b) refers to patients who have been treated with immune checkpoint inhibitors or corticosteroids to reduce intracranial pressure in the past 1 month

### Safety analyses of phase I trial

The course of atomization EGCG is 7 days, which can be increased to a maximum of 14 days according to the wishes of the patients. However, only 7-day changes were selected for safety and efficacy evaluation. The EGCG solution was well tolerated. No adverse events of > Grade 1 was found in the whole population. Bucking but quickly recover was observed after EGCG administration in one patient (1760µmol/L). Three more patients were enrolled at this level and no more patient experienced toxicities of EGCG. In the fourth dose echelon, two more patients showed adverse events of nausea and upset stomach. The dose escalation stopped at 8817 µmol/L. And the maximum dose at which no adverse events were observed of Phase I trial (5878 µmol/L) was defined as a recommended dose for the Phase II trial.

### Safety and efficacy analysis of phase II trial

**Fig. 2:**
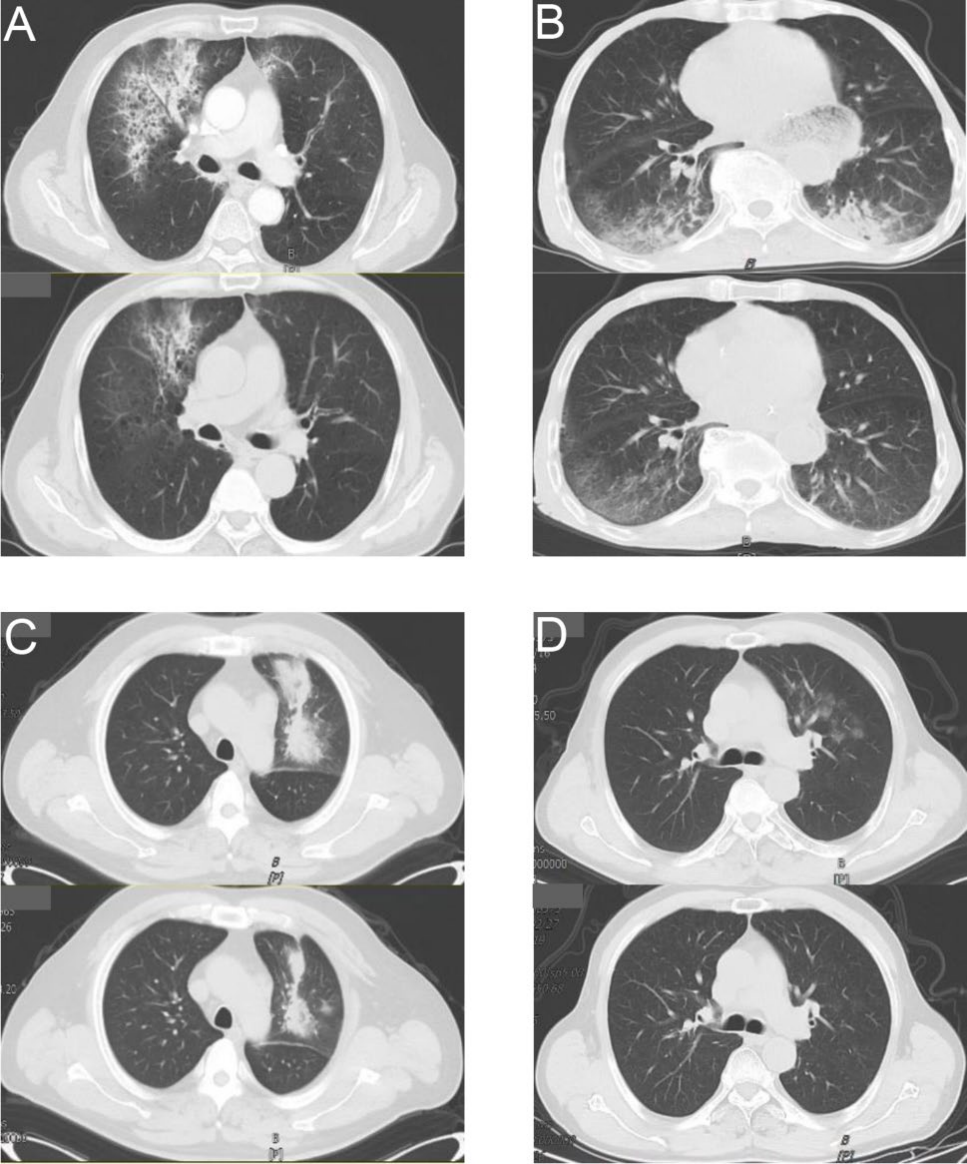
Optimal CT changes in several patients with COVID-19 pneumonia. Two CT slides of four cancer patients are shown respectively within 1-week EGCG treatment. The top is the baseline and the bottom is significantly absorbed and dissipated improved chest imaging

In the phase II clinical trial, we further verified the safety of oxygen-driven atomization inhalation of EGCG. Among them, three of the patients failed to complete the full course of treatment because of the aggravation of chest tightness and irritating dry cough in the second time and expectoration at the third day. We confirmed the efficacy of EGCG through CT imaging findings. After one week of medication, the re-evaluation CT showed improvement in 22 patients(56.4%), one of the patients had almost complete absorption of pneumonia (without steroids, pure EGCG inhalation treatment)(CT condition was shown in Fig. 2D). Unfortunately, 7 patients (17.9%) failed to control the progression of inflammation, including 5 patients with enlarged primary inflammatory lesions and 2 patients with improved primary inflammatory lesions but new lesions. There was no change in CT re-evaluation for 10 patients (25.6%). Thus, the results of our Phase II trial showed that the non-progression rate of 82.0% (32/39) and an improvement rate of 56.4% (22/39). The changes in pneumonia in several representative patients are shown in Fig. 2. However, there was no significant difference in inflammation-related laboratory indexes (white blood cell, lymphocyte, IL-6, ferritin, C-reactive protein and lactate dehydrogenase) before and after treatment.

## Discussion

At present, few studies have been carried out on cancer patients after COVID-19 infection, and as a vulnerable group that can’t be ignored, more attention should be paid to their management. For our information, this is the first phase I/II clinical trial to evaluate the anti-inflammatory effect of EGCG in cancer patients with COVID-19 pneumonia. In this Phase I/II trial, we found that atomized EGCG for cancer patients with COVID-19 was well tolerated. We conducted a dose-escalating study for atomized EGCG (1760µmol/L to 8817µmol/L), only nausea and stomach discomfort were observed in the highest dose. Therefore, the recommended dose of EGCG in the phase II is defined as the highest concentration without a dose-limiting toxicity (DLT) observed (5878µmol/L). And this study met its primary endpoint of showing a favorable safety profile and a significant imaging improvement rate and non-progression rate of severe pneumonia, which might have potentially resulted in lesser duration of oxygen support, lower ICU admissions and earlier anti-tumor treatment.

It has been reported that most of the adverse events of EGCG treatment are related to gastrointestinal disorders, mainly nausea, abdominal pain or discomfort, diarrhea, dyspepsia and elevated liver enzymes [20], which was consistent with the adverse events of two patients when we administered the highest dose. It undeniable that different routes of administration may result in varying concentrations of EGCG reaching target organs and thus affecting efficacy. The therapeutic potential of orally administered EGCG is limited by poor gastrointestinal absorption and bioavailability [21]. Even with intravenous administration, EGCG can partially degrade before reaching target tissues [22]. Therefore, we consider local administration as an alternative approach. Our past studies have shown promising efficacy of mucosal or topical administration in the treatment of radiation-induced esophagitis [19] and protection against radiation-induced dermatitis (RID) [18]. Previous studies have confirmed that nebulized inhalation of EGCG exhibits good tolerability and anti-infection activity against bacterial lung infections [23]. Inhaled drug delivery could directly act on the target organs, avoid the first-pass effect of the liver, improve the bioavailability of the lungs and reduce systemic side effects. Sahin et al. have also proposed that inhalation administration is the most promising method for treating COVID-19 [24]. A recent study [25] enrolled 10 patients with COVID-19 infection who received EGCG nebulized inhalation and capsule administration (total EGCG: 595 mg). The dose was much higher than our concentration, but showed well tolerance and a significant decrease in inflammatory markers. Based on the above evidence, we chose nebulized inhalation of EGCG as a promising method.

Optimal therapy should be based on the pathogenesis SARS-CoV-2 infections, particularly those with known co-morbidities. SARS-CoV-2 infection begins with infection of ciliated cells in the upper respiratory tract. Due to dysregulated or mistimed immune responses, particularly poor type I and type III interferon (IFN) responses, the virus spreads down the bronchi tracheal tree to the alveoli, leading to infection of alveolar type 2 (AT2) cells and endothelial activation. Additionally, both the epithelium and the endothelium are induced into a “leaky state”, promoting inflammation and coagulation; immune cells such as monocytes or macrophages and neutrophils are attracted, further amplifying pro-inflammatory and/or pro-fibrotic responses [26]. Uncontrolled inflammation ultimately leads to severe pneumonia of COVID-19 or ARDS with diffuse alveolar damage. In addition, Siddiqi and Mehra proposed the three stages of COVID-19, stage I lasts about a week for early viral infection, which is characterized by symptoms of an upper respiratory tract infection [27]. Stage II is the lung phase, which involves early host inflammatory response and could be appropriately treated with anti-inflammatory therapy [27]. Stage III is the hyperinflammation stage that often leads to acute respiratory distress syndrome (ARDS). The progressive and persistent pathophysiologic features of severe COVID-19 are primarily driven by inflammatory responses [28]. After COVID-19 infection, pneumonia was observed in 75% of the patients and approximately 20% develop severe stages [1, 29]; severe COVID-19 may also result in extrapulmonary manifestations such as gastrointestinal symptoms and acute cardiac, kidney and liver injury [30]. Our study included cancer patients with moderate pneumonia and gave effective anti-inflammatory interventions during the host inflammatory response stage to avoid COVID-19 progression.

The most effective anti-inflammatory drugs employed so far in severe COVID-19 were only cytokine-directed biological agents [31] and corticosteroids [29, 32]. As is known to all, corticosteroids frequently used for severe and critical COVID-19 who require ventilation, and prevent or mitigate these deleterious effects by regulating the strong anti-inflammatory effects of cytokines release [33]. However, a meta-analysis reported that corticosteroid use was associated with increased mortality in patients with coronavirus or other viral pneumonia [34], which may be related to adverse effects such as bacterial and fungal infections caused by immunosuppression, hyperglycemia and hypothalamus-pituitary-adrenal axis inhibition. Severe COVID-19 is not only associated with excessive inflammation, but also with immune dysfunction. Patients with immune dysfunction caused by COVID-19 or corticosteroid therapy are more likely to develop secondary pulmonary infection and increase the risk of death [32, 35]. Therefore, for people at high risk of severe COVID-19, cheap, low-toxic and effective anti-inflammatory drugs are urgently needed.

Recently, in the diagnosis and treatment protocol for novel coronavirus pneumonia trial version 1–10 and other treatment guidelines in China, more than 250 prescriptions and patent medicines, including natural products (with Western medicine), were added to the recommended drugs for the prevention and treatment of COVID-19 [36]. Experts have used natural products, especially flavonoids, as key compounds to reduce the incidence of severe or critical events, improve clinical recovery, and help alleviate symptoms such as cough or fever [36]. Epigallocatechin-3-gallate (EGCG), one of the representative flavonoid components could enable resistance against COVID-19 by regulating inflammatory, antiviral, and immune responses, and repairing tissue injury. Notably, in the immune system, most cytokine receptors, including IL-6, IL-10, IL-21, and IL-23, can activate the important nuclear factor STAT3 that regulates immune and autoimmune response [37]. In COVID-19 hyperinflammation condition, high levels of IL-6 seem to be the main prognostic factor for poorer outcomes. The use of tocilizumab that can inhibit IL-6 signaling and block disease progression. Similarly, EGCG may also be promising as it is a potent blocker of the STAT3 pathway [12]. EGCG also can block the activation of NF-κB to controls the expression of many pro-inflammatory cytokines, including IL-1β, TNF-α, IL8, IL-6, all of which are induced in COVID-19 [38–40].

Based on the anti-inflammatory effects of EGCG, previous studies have proposed that EGCG counteracts hyper-inflammation growing in COVID-19 and prevents further aggravation leading to inflammatory markers decline [12]. However, our trial was not detected significant changes in all laboratory inflammation-related outcomes. Systemic inflammatory reflection (SIR) is recognized as the 7th symbol of cancer which is closely associated with the occurrence and progression of malignancies [41]. Increased immunocyte in the tumor microenvironment have been reported to prompt the tumor growth and metastasis by releasing chemokines and cytokine [42]. Therefore, we speculate that the inflammatory microenvironment of tumor patients may be responsible for the non-significant changes in the inflammatory indexes.

No matter what stage the tumor is in, delaying oncologic treatment may lead to disease progressions and result in worse survival outcomes. Cortiula et al. suggest that advanced tumor patients with no suggestive symptoms of COVID-19, should keep receiving planned chemotherapy or radiotherapy treatment, without unnecessary delays [43]. However, for patients who continue to receive immunotherapy or radiotherapy, when obvious inflammation of the lungs is not absorbed, partial clinicians do not dare to use anti-tumor therapy rashly, but suggest the treatment of pneumonia first. Additionally, a median time of 15 days was reported for mild to moderate cases and 3–6 weeks for severe or critical patients’ conditions from the onset to clinical recovery. Some patients with COVID-19 who seem stable or on the mend can suddenly become critically ill during the second week, called “second-week crash [44]. Moreover, looking back at past outbreaks and pandemics, thorough eradication of a pathogen is rarely achieved. Even if the new SARS-CoV-2 disappears for a while, we might potentially see it coming back as an endemic cause of seasonal pneumonia [43]. Taking all of these factors into account, EGCG can be considered a potential natural supplement and popularize to treat COVID-19 pneumonia and control the progression.

Despite the obvious advantages, the limitations of the trial should also be emphasized. Firstly, our sample size of single-center was small and the trial only enrolled cancer patients, not include non-cancer population. As the COVID-19 pandemic is a sudden and temporary event, cancer patients who are admitted to hospital for anti-tumor treatment are often out of the SARS-CoV-2 infection period. CT shows that the pulmonary inflammation has subsided, meanwhile, the number of recruited severe or critically ill patients with COVID-19 pneumonia is also limited. Additionally, considering the urgent clinical needs and potential benefits are far below than cancer patients, as well as their lower compliance, feasibility and cost of the trial, we failed to enrolled the non-tumor population. The sample size and population need to be further optimized to validated the safety and efficacy of EGCG. Secondly, although we enrolled patients in the epidemic strain of Omicron in China, it is a pity that due to the biosafety level of laboratory (P2), we did not accurately detect the strain of each patient. In the future, we will continue to subdivide the COVID-19 strain in biosafety level 3 (P3) laboratories and explore whether the strain types are related to its efficacy. Lastly, this trial did not include a control group, therefore, reducing the generalizability of our results. Because there is no approved effective conventional treatment other than corticosteroids for patients with severe or critical COVID-19. Given the ethical considerations of protecting the maximum benefits of participants and the objectives (rapid discovery of EGCG safety and efficacy) of this investigator-initiated clinical trial, no control group was recruited for this study. Multicenter randomized phase III trials will continue to be conducted in the future to assessed the effect of EGCG.

## Conclusion

Aerosol inhalation of EGCG is well tolerated, and preliminary investigation suggests that EGCG may be effective in controlling the progress and promoting improvement of SARS-CoV-2-induced pneumonia in cancer patients with high risk factors of severe COVID-19. This favorable result provides a strong evidence for the future multi-center, randomized phase III clinical trial.

### Electronic supplementary material

Below is the link to the electronic supplementary material.

Supplementary Material 1

## Data Availability

No datasets were generated or analysed during the current study.

## Abbreviations

SARS-CoV-2: Severe Acute Respiratory Syndrome Coronavirus 2
COVID-19: Corona Virus Disease 2019
NSCLC: non-small cell lung cancer
EGCG: epigallocatechin-3-gallate
NCI CTCAE: National Cancer Institute for Common Terminology Criteria for Adverse Events
MTD: maximally tolerated dose
SD: Standard Deviation
CONSORT: Consolidated Standards of Reporting Trials
AJCC: American Joint Committee on cancer
RID: radiation-induced dermatitis
AT2: alveolar type 2
STAT3: signal transducer and activator of transcription 3
IL: interleukin
SIR: systemic inflammatory reflection
PD-L1: Programmed Cell Death-Ligand 1
PD-1: Programmed Death-1
ECOG-PS: Eastern Cooperative Oncology Group Performance Status
