# Supplementary material for: Phase I/II clinical trial of efficacy and safety of EGCG oxygen nebulization inhalation in the treatment of COVID-19 pneumonia patients with cancer
Source: BMC Cancer. 2024 Apr 17;24:486. doi: 10.1186/s12885-024-12228-3 (PMC11022442; doi:10.1186/s12885-024-12228-3)
Supplement: Supplementary file 1 — Supplementary Material 1 [file 12885_2024_12228_MOESM1_ESM.docx]

**Phase I/II clinical trial of efficacy and safety of EGCG oxygen nebulization inhalation in the treatment of COVID-19 pneumonia patients with cancer**

| **Study group** | |
| --- | --- |
| Study Chair  Xiangjiao Meng, MD, PhD  Department of Radiation Oncology  Shandong Cancer Hospital and Institute 440 Jiyan Road  Jinan, Shandong 250117  86-531-67626819  mengxiangjiao@sina.com | Hanxi Zhao, MD, PhD  Department of Radiation Oncology  Shandong Cancer Hospital and Institute 440 Jiyan Road  Jinan, Shandong 250117  86-531-67626995/Fax 86-531-67626141  zhx87520052@163.com |
| Ligang Xing, MD, PhD  Shandong Cancer Hospital  and institute/Shandong Key Laboratory of Radiation Oncology  440 Jiyan Road  Jinan, Shandong 250117  86-531-67626819/Fax 86-531-67626819  xinglg@medmail.com.cn | Jinming Yu, MD, PhD  Shandong Cancer Hospital  and institute/Shandong Key Laboratory of Radiation Oncology  440 Jiyan Road  Jinan, Shandong 250117  86-531-67626919/Fax 86-531-87984729  sdyujinming@126.com |
| Xiaoyan Yin, MD  Department of Radiation Oncology  Shandong Cancer Hospital and Institute 440 Jiyan Road  Jinan, Shandong 250117  86-531-67626995  yinxiaoyan07@163.com | Wanqi Zhu, MD  Department of Radiation Oncology  Shandong Cancer Hospital and Institute 440 Jiyan Road  Jinan, Shandong 250117  86-531-67626995/Fax 86-531-67626141  zhuwanqi@sdfmu.edu.cn |
| Xianguang Zhao, MD  Department of Radiation Oncology  Shandong Cancer Hospital and Institute 440 Jiyan Road  Jinan, Shandong 250117  86-531-67626995  zhaoxg2011@163.com | Xiaolin Li, MD, PhD  Department of Radiation Oncology  Shandong Cancer Hospital and Institute 440 Jiyan Road  Jinan, Shandong 250117  86-531-67626995  daisylinjinan@163.com |
| Kaikai Zhao, MD, PhD  Department of Radiation Oncology  Shandong Cancer Hospital and Institute 440 Jiyan Road  Jinan, Shandong 250117  86-531-67626995  abcdkaikai35@126.com | Liyang Jiang, MD, PhD  Department of Radiation Oncology  Shandong Cancer Hospital and Institute 440 Jiyan Road  Jinan, Shandong 250117  86-531-67626995  lorry@msn.com |
| Hong Zhao, MD, PhD  Department of Radiation Oncology  Shandong Cancer Hospital and Institute 440 Jiyan Road  Jinan, Shandong 250117  86-531-67626995  zhaohong-vivi@whu.edu.cn | Xin Wang, MD, PhD  Department of Radiation Oncology  Shandong Cancer Hospital and Institute 440 Jiyan Road  Jinan, Shandong 250117  86-531-67626995  wangoncologist@126.com |
| Xiaoyong Tang, MD, PhD  Department of Medical Oncology  Shandong Cancer Hospital and Institute 440 Jiyan Road  Jinan, Shandong 250117  86-531-67626995  cot163@163.com | Guangjian Yang, MD, PhD  Department of Medical Oncology  Shandong Cancer Hospital and Institute 440 Jiyan Road  Jinan, Shandong 250117  86-531-67626995  yekong0806@126.com |
| Yuanyuan Yan, MD  Department of Radiology  Shandong Cancer Hospital and Institute 440 Jiyan Road  Jinan, Shandong 250117  86-531-67626721  once_99@163.com |  |

**TABLE OF CONTENTS**

Schema

Eligibility Checklist

1.0 Introduction

2.0 Objectives and Endpoints

3.0 Patient Selection

4.0 Study Design

5.0 Drug Administration

6.0 Patient Assessments

7.0 Statistical Considerations

References

Appendix I – COVID-19 infection diagnosis and treatment plan (Version 10) (issued by National Health Commission of PRC)

Appendix II -Sample Consent Form (In Chinese)

**Phase I/II clinical trial of efficacy and safety of EGCG oxygen nebulization inhalation in the treatment of COVID-19 pneumonia patients with cancer**

**SCHEMA**

| Patients confirmed COVID-19 | **R**  **E**  **G**  **I**  **S**  **T**  **E**  **R** | Patients who meet all criteria were randomly assigned | **E**  **S**  **C**  **A**  **L**  **A**  **T**  **I**  **O**  **N** | I: EGCG for 1760, 3520, 5878 and 8817 umol/L per dose  II: EGCG for the highest concentration in the absence of MTD observation | Evaluation after treatment and follow-up for one month |
| --- | --- | --- | --- | --- | --- |

**Inclusion criteria**

Patients fulfilling all the following criteria will be eligible:

🞄Age ≥ 18 years

🞄Diagnosed malignant tumors by pathology or cytology

🞄COVID-19 with a confirmed reverse transcription-polymerase chain reaction (RT-PCR) or antigen test result for SARS-CoV-2

🞄Moderate pneumonia, according to COVID-19 Infection Diagnosis and Treatment Plan (version 10), issued by the National Health Commission of the PRC

🞄Pulmonary function of the patient can be treated with aerosol inhalation for 5-7 days

**Exclusion criteria**

🞄Current or recent progresses rapidly and may develop into a critical illness with coronavirus in a short period of time

🞄Pregnancy or breastfeeding

🞄Long term corticosteroids therapy at a dose of 0.5mg/kg/d or higher

🞄Caused by other viruses such as cytomegalovirus, as well as pneumocystis pneumonia, pulmonary edema, aspiration pneumonia and acute interstitial pneumonia

🞄Any condition including medical, emotional, psychiatric, or logistical that, in the opinion of the Investigator would preclude the participant from adhering to the protocol or would increase the risk associated with study participation

🞄Need systemic use of immune suppressive agents

🞄Allergic to EGCG

🞄With other diseases that may affect the timely completion of the treatment plan

**Elimination criteria**

🞄Not take the drugs as prescribed during the trial and failed to complete 80% of the planned medication

🞄Not cooperate with the follow-up

🞄Supervising physician believes that interrupting the study is beneficial for the patient

**Eligibility Checklist**

**Case#**

_______(Y)1.Being or has been confirmed COVID-19 infection

_______(Y)2.Pathologically or cytologically proven malignant tumors

_______(Y)3.Is age≥18 eighteen years?

_______(Y)4.Dose the patient’s CT show the characteristic manifestations of novel coronavirus infection pneumonia?

_______(Y)5.Clinical evaluation as moderate pneumonia (persistent high fever for more than 3 days or / and cough, shortness of breath, etc., the respiratory frequency (RR) less than 30 beats / min, and the oxygen saturation > 93% when inhaling air at rest)

_______(N)6. Does the patient combine with other diseases that affect treatment (such as cerebral infarction, persistent atrial fibrillation, etc)?

_______(N)7. Does the patient use immunosuppressive drugs for a long time?

_______(N)8. Is the patient pregnant or lactational?

_______(N)9. Is the patient on any other study?

_______(N)10. Does the patient have any mental incompetence, including psychological or addictive disorders which would preclude completion of questionnaires?

**The following questions will be asked at Study Registration:**

________1. Name of institutional person registering this case?

______(Y)2. Has the Eligibility Checklist been completed?

______(Y)3. In the opinion of the investigator, is the patient eligible?

________4. Date informed consent signed

________5. Patient’s Initials (First Middle Last)

________6. Patient ID

________7. Verifying Physician

________8. Date of Birth

________9. Ethnicity

________10. Zip Code

________11. Method of Payment

________12. Treatment Start Date

________13. Treatment Assignment

Completed by Date

**1.0 INTRODUCTION**

SARS-Cov-2 is a type of novel coronavirus that has caused severe acute respiratory syndrome spread around the world since the end of 2019^1, 2.^ Patients with characteristics as older age, smoking history or complications such as cardiovascular disease, diabetes, obesity, and cancer are at high risk for severe COVID-19 and related severe events^1, 3, 4^. It has been described that compared with patients without those condition, patients with prespecified coexisting disease were about approximately twice as likely to exacerbate to severe COVID-19 and five-fold high mortality rate^5, 6^. Undoubtably, cancer patients were more vulnerable to SARS-CoV-2 and show more deteriorating conditions and poor outcomes than individuals without cancer because of their systemic immunosuppressive state caused by the malignancy and anticancer treatments^7-9^. Most importantly, patients with cancer were observed to be older, more likely to have a history of smoking and more severe baseline CT manifestation^8^,which possess more risk factors for severe COVID-19.

SARS-Cov-2 induces the uncontrolled release of excessive inflammatory cytokines by host immune response^10^, resulting in mild to severe pneumonia, acute lung injury and even hypoxic respiratory failure. This over-production of pro-inflammatory cytokines induces more damage to the host cells than the one induced by SARS-CoV-2 as pathogen invader^11^. Although Paxlovid and Nirmatrelvir or other specific therapeutics could reduce viral load and the incidence of severe covid-19 in the early stage of viral infection, the inhibition of inflammatory response, elimination of pulmonary inflammation and repair of injury in patients with COVID-19 are also the important consideration in clinic^12^. However, for hyper-inflammation, there are currently no effective and low side-effect drugs to curb the development of pneumonia except corticosteroids or cytokine-directed biological agents.

Epigallocatechin-3-gallate (EGCG), the major and most highly bioactive constituent in green tea, has strong anti-inflammatory, anti-oxidant, antiviral and even antitumor activities ^13, 14^.Some studies found EGCG can downregulate the expression of inflammatory mediators and signal transduction pathways by acting on signal transducers and activators of transcription (STAT) 1/3 and nuclear factor kappa-light-chain-enhancer of activated B cells (NF-κB) transcription factors^11^ ,which could be considered as a potential nature immune homeostasis agent to counteract hyper-inflammation existing in COVID-19.

In our previous studies^15-18^, we found that EGCG can prevent and cure radiation-induced normal tissue damage in tumor patients with high safety and potent anti-inflammatory competence. Other clinical studies also documented favorable security of EGCG. Based on these encouraging results, we conducted this phase I-II clinical trial to investigate the possible role of EGCG aerosol inhalation in the treatment of interstitial pneumonia in tumor patients infected with novel coronavirus.

**2.0 OBJECTIVES AND ENDPOINTS**

**2.1** **Primary Objective**

**2.1.1 To evaluate the efficacy of EGCG in controlling moderate pneumonia resulting from COVID-19, including promoting remission of pneumonia or inhibiting the progression of pneumonia**

**2.2** **Secondary Objective**

**2.2.1 To evaluate the safety of nebulized inhalation of EGCG in the treatment of COVID-19 pneumonia in cancer patients.**

**2.3 Endpoints**

**2.3.1 Primary efficacy endpoint: inflammatory changes on CT and changes in laboratory inflammatory indexes at Day 0 and Day 7**

**2.3.2 Secondary safety endpoint: types and incidence of adverse events**

**3.0 PATIENT SELECTION**

- 1. **Conditions for Patient Eligibility**

3.1.1Obtain informed consent signed by patients

3.1.2Patients with malignant tumors confirmed by pathology or cytology

3.1.3Age ≥ 18 years

3.1.4COVID-19 with a confirmed positive polymerase chain reaction or antigen test result for SARS-CoV-2

3.1.5Patients with moderate pneumonia, according to COVID-19 Infection Diagnosis and Treatment Plan (version 10), issued by the National Health Commission of the PRC

3.1.6Pulmonary function of the patient can be treated with aerosol inhalation for 5-7 days

3.1.7Normal renal function: creatinine clearance rate ≥50ml / min

3.1.8Normal liver function: bilirubin level ≤1.5 times the upper limit of normal value

3.1.9Normal electrocardiogram

## 3.2 Conditions for Patient Ineligibility

3.2.1Pregnant or lactating women, those who are in the reproductive period and do not take effective contraceptive measures

3.2.2With mental illness or neurological disorders and are unable to speak clearly about the treatment response (such as sequelae of cerebrovascular accident)

3.2.3With severe heart, liver, kidney and other organ diseases or diabetes who are not expected to complete the treatment plan

3.2.4Allergic to EGCG

3.2.5With other diseases that may affect the timely completion of the treatment plan

3.2.6Patients who do not understand the trial requirements or may not comply with the trial requirements

3.2.7Current or recent progresses rapidly and may develop into a critical illness with coronavirus in a short period of time

3.2.8Long term corticotherapy at a dose of 0.5mg/kg/d or higher

3.2.9Caused by other viruses such as cytomegalovirus, as well as pneumocystis pneumonia, pulmonary edema, aspiration pneumonia and acute interstitial pneumonia

3.2.10Need systemic use of immune suppressive agents

**4.0 STUDY DESIGN**

Based on the previous research of our group, this project adopted the design scheme of phase I-II clinical study, in which phase I adopted the improved Fibonacci method, four dose levels for EGCG were defined as following: 1760, 3520, 5878 and 8817 umol/L per dose. Dose escalation proceeded according to a standard phase I design with three patients initially treated on each tier. If, on any dose tier of EGCG, two of three patients or two of six patients experienced toxicity due to EGCG, dose escalation of EGCG would cease. The patient inhaled 10ml EGCG by atomization three times a day. From the beginning of the diagnosis and 7 days after the signing of the informed consent form, the medication can be continued according to the wishes of the patients, but the total medication time is not more than 14 days. The primary end point of the phase I study was the safety of EGCG. The CTCAE5.0 version was used to evaluate the safety of EGCG for this kind of patients, and the secondary end point was the effectiveness of EGCG. The evaluation method was the changes of imaging examination (chest CT) before and after treatment. In the II clinical study, the dose determined by phase I was a non-random, single-arm trial. The main evaluation end point: the changes of imaging examination (chest CT) before and after treatment. Secondary evaluation end point: 1 further evaluate the security of EGCG. (2) improvement of general condition. (3) the changes of laboratory testing indexes.

## DRUG ADMINISTRATION

- 1. **Product Information: EGCG**
     1. Epigallocatechin-3-gallate (EGCG), the major catechin found in great tea, had the Potential anti-inflammatory ability in vitro and in vivo. Oral pills of green tea polyphenol products are available commercially as dietary supplements. It is a non- prescription product. Nebulized inhalation of EGCG has also been reported in previous studies^19, 20^.
     2. Supply: Purchased from Ningbo Hepu Biotechnology Co., Ltd.
     3. Ingredients: EGCG (purity ≥98%) freshly dissolved in 0.9% saline solution.

## Application

- - 1. EGCG (purity 98%) was purchased from Ningbo Hepu Biotechnology Co., Ltd. and its solution was freshly prepared. The application of EGCG solution was initiated from Day1, that is the same day the informed consent form was signed. Then, the EGCG solutions were uniformly atomized inhaled 10ml three times a day .For patients with obvious respiratory symptoms such as cough, spittoon and shortness of breath, ambroxol and other non-glucocorticoid and non-anti-inflammatory drugs can be inhaled.

## 6.0 ASSESSMENTS

**6.1 Study Parameters**

**6.1.1Baseline evaluation before treatment**

| Project | | Time before treatment |
| --- | --- | --- |
| Informed consent form | sign | 0-1day |
| Inclusion or exclusion criteria | verify | 0-1day |
| Diagnosis and staging of primary tumor | AJCC stage，2009，version 7 | 0-1day |
| Past medical history | Including concomitant diseases | 0-1day |
| Physical | Weight and height, etc. | 0-1day |
| Vital signs | temperature, pulse, breathing (resting state), blood pressure, oxygen saturation (non-inhaling oxygen) | 0-1day |
| ECOG PS |  | 0-1day |
| Imaging assessment | Chest CT | 1-3day |
| Electrocardiogram |  | 0-1day |
| Hematological  examination | D-dimer, liver / renal function, lactate dehydrogenase, C-reactive protein (CRP). Ferritin, procalcitonin (PCT), absolute value of lymphocytes, interleukin-6 (IL-6) | 0-1day |

All questionnaires and instructions will be reviewed with the patient prior to enrollment.

**6.1.2Evaluation in the course of treatment**

| **Project** | | **Time** |
| --- | --- | --- |
| Vital signs | temperature, pulse, breathing (resting state), blood pressure, oxygen saturation (non-inhaling oxygen) | daily |
| Adverse Events | The observation period for collecting adverse events was 7 days from the first administration to the last administration. Serious adverse events should be carried out in accordance with the provisions of the plan. Record the accompanying treatment of adverse events. | daily |

**6.1.3Evaluation after the completion of treatment**

| **Project** | | **Time after treatment** |
| --- | --- | --- |
| Vital signs | temperature, pulse, breathing (resting state), blood pressure, oxygen saturation (non-inhaling oxygen) | 0-1day |
| ECOG PS |  | 0-1day |
| Imaging assessment | Chest CT | 0-1day |
| Electrocardiogram |  | 0-1day |
| Hematological  examination | D-dimer, liver / renal function, lactate dehydrogenase, C-reactive protein (CRP). Ferritin, procalcitonin (PCT), absolute value of lymphocytes, interleukin-6 (IL-6) | 0-1day |

The patients were followed up according to NCCN guideline after drug finished. Chest CT images were acquired after 7day of the treatment.

**6.2 Radiology assessment**

CT evaluation criteria refer to Chen Yousan's chest CT score criteria, the common SARS-CoV-2 infectious CT performance: the initial CT evaluation showed multiple small ground glass nodules with patchy lesions distributed along the bronchovascular bundle; central consolidation with surrounding ground glass density shadows, single or multiple central ground glass nodules or patches, unilateral or bilateral consolidation; multiple patchy, segmental, or large ground glass density shadows or interlobular septal thickening or consolidation in the subpleural or multi center distribution of both lungs. We classified the treatment outcomes of COVID-19-related pneumonia into three categories. Improvement: the re-evaluation of CT showed that the range of inflammation was limited to the initial CT imaging, and showed consolidation, absorption or fibrosis, or even disappeared completely. Deterioration: the scope of the lesion exceeds the initial CT image or new inflammatory lesions appear. Stable is between improvement or deterioration. Two senior imaging physicians and one clinical physician independently interpret and verify the interpretation opinions on site.

**6.3 Safety and Inflammation indexes Assessments**

Safety assessments were performed according to the National Cancer Institute Common Terminology Criteria for Adverse Events, version 5.0. Some adverse events and the changes of Inflammation or other hematological indexes were monitored using laboratory tests, such as complete blood cell count, chemistry profile, and liver function tests.

## 7.0 STATISTICAL CONSIDERATIONS

**7.1 Analysis Methods**

The major analysis will be undertaken when all patients have completed the treatment. The usual components of this analysis are:

🞄Tabulation of all cases entered and any excluded from the analysis with the reasons for such exclusions;

🞄Distribution of pretreatment characteristics;

🞄Observed results with respect to the study endpoints: the safety assessments were recorded daily from the start of administration to its completion. The efficacy analysis set included patients who completed the composite assessment by the investigators. Statistical analyses were performed using SPSS Statistics for Windows, version 26.0. Measurement data are expressed as mean and SD and analyzed by t test. All the P values are 2-sided.

**REFERENCE**

1. Wiersinga WJ, Rhodes A, Cheng AC, Peacock SJ, Prescott HC. Pathophysiology, Transmission, Diagnosis, and Treatment of Coronavirus Disease 2019 (COVID-19): A Review. *Jama*. Aug 25 2020;324(8):782-793. doi:10.1001/jama.2020.12839

2. Wu F, Zhao S, Yu B, et al. A new coronavirus associated with human respiratory disease in China. *Nature*. Mar 2020;579(7798):265-269. doi:10.1038/s41586-020-2008-3

3. Kim L, Garg S, O'Halloran A, et al. Risk Factors for Intensive Care Unit Admission and In-hospital Mortality Among Hospitalized Adults Identified through the US Coronavirus Disease 2019 (COVID-19)-Associated Hospitalization Surveillance Network (COVID-NET). *Clin Infect Dis*. May 4 2021;72(9):e206-e214. doi:10.1093/cid/ciaa1012

4. Zheng Z, Peng F, Xu B, et al. Risk factors of critical & mortal COVID-19 cases: A systematic literature review and meta-analysis. *J Infect*. Aug 2020;81(2):e16-e25. doi:10.1016/j.jinf.2020.04.021

5. Thakur B, Dubey P, Benitez J, et al. A systematic review and meta-analysis of geographic differences in comorbidities and associated severity and mortality among individuals with COVID-19. *Sci Rep*. Apr 20 2021;11(1):8562. doi:10.1038/s41598-021-88130-w

6. Hammond J, Leister-Tebbe H, Gardner A, et al. Oral Nirmatrelvir for High-Risk, Nonhospitalized Adults with Covid-19. *N Engl J Med*. Apr 14 2022;386(15):1397-1408. doi:10.1056/NEJMoa2118542

7. Sica A, Massarotti M. Myeloid suppressor cells in cancer and autoimmunity. *J Autoimmun*. Dec 2017;85:117-125. doi:10.1016/j.jaut.2017.07.010

8. Liang W, Guan W, Chen R, et al. Cancer patients in SARS-CoV-2 infection: a nationwide analysis in China. *Lancet Oncol*. Mar 2020;21(3):335-337. doi:10.1016/s1470-2045(20)30096-6

9. Dai M, Liu D, Liu M, et al. Patients with Cancer Appear More Vulnerable to SARS-CoV-2: A Multicenter Study during the COVID-19 Outbreak. *Cancer Discov*. Jun 2020;10(6):783-791. doi:10.1158/2159-8290.Cd-20-0422

10. Dorward DA, Russell CD, Um IH, et al. Tissue-Specific Immunopathology in Fatal COVID-19. *Am J Respir Crit Care Med*. Jan 15 2021;203(2):192-201. doi:10.1164/rccm.202008-3265OC

11. Menegazzi M, Campagnari R, Bertoldi M, Crupi R, Di Paola R, Cuzzocrea S. Protective Effect of Epigallocatechin-3-Gallate (EGCG) in Diseases with Uncontrolled Immune Activation: Could Such a Scenario Be Helpful to Counteract COVID-19? *Int J Mol Sci*. Jul 21 2020;21(14)doi:10.3390/ijms21145171

12. Ozturk T, Talo M, Yildirim EA, Baloglu UB, Yildirim O, Rajendra Acharya U. Automated detection of COVID-19 cases using deep neural networks with X-ray images. *Comput Biol Med*. Jun 2020;121:103792. doi:10.1016/j.compbiomed.2020.103792

13. Kada T, Kaneko K, Matsuzaki S, Matsuzaki T, Hara Y. Detection and chemical identification of natural bio-antimutagens. A case of the green tea factor. *Mutat Res*. Jun-Jul 1985;150(1-2):127-32. doi:10.1016/0027-5107(85)90109-5

14. Chakrawarti L, Agrawal R, Dang S, Gupta S, Gabrani R. Therapeutic effects of EGCG: a patent review. *Expert Opin Ther Pat*. Aug 2016;26(8):907-16. doi:10.1080/13543776.2016.1203419

15. Zhao H, Jia L, Chen G, et al. A prospective, three-arm, randomized trial of EGCG for preventing radiation-induced esophagitis in lung cancer patients receiving radiotherapy. *Radiother Oncol*. Aug 2019;137:186-191. doi:10.1016/j.radonc.2019.02.022

16. Zhao H, Zhu W, Xie P, et al. A phase I study of concurrent chemotherapy and thoracic radiotherapy with oral epigallocatechin-3-gallate protection in patients with locally advanced stage III non-small-cell lung cancer. *Radiother Oncol*. Jan 2014;110(1):132-6. doi:10.1016/j.radonc.2013.10.014

17. Zhao H, Zhu W, Zhao X, et al. Efficacy of Epigallocatechin-3-Gallate in Preventing Dermatitis in Patients With Breast Cancer Receiving Postoperative Radiotherapy: A Double-Blind, Placebo-Controlled, Phase 2 Randomized Clinical Trial. *JAMA Dermatol*. Jul 1 2022;158(7):779-786. doi:10.1001/jamadermatol.2022.1736

18. Li X, Xing L, Zhang Y, et al. Phase II Trial of Epigallocatechin-3-Gallate in Acute Radiation-Induced Esophagitis for Esophagus Cancer. *J Med Food*. Jan 2020;23(1):43-49. doi:10.1089/jmf.2019.4445

19. Haddad F, Mohammed N, Gopalan RC, Ayoub YA, Nasim MT, Assi KH. Development and Optimisation of Inhalable EGCG Nano-Liposomes as a Potential Treatment for Pulmonary Arterial Hypertension by Implementation of the Design of Experiments Approach. *Pharmaceutics*. Feb 6 2023;15(2)doi:10.3390/pharmaceutics15020539

20. Vidigal PG, Müsken M, Becker KA, et al. Effects of green tea compound epigallocatechin-3-gallate against Stenotrophomonas maltophilia infection and biofilm. *PLoS One*. 2014;9(4):e92876. doi:10.1371/journal.pone.0092876

Appendix I

COVID-19 infection diagnosis and treatment plan

(Version 10)

CONTENTS

[1. Pathogenic Characteristics 19](#_Toc157440753)

[2. Epidemiological Characteristics 20](#_Toc157440754)

[2.1 Infection Source 20](#_Toc157440755)

[2.2 Transmission Route 20](#_Toc157440756)

[2.3 Susceptible Population 20](#_Toc157440757)

[3. Prevention 21](#_Toc157440758)

[3.1 COVID-19 Vaccine Inoculation 21](#_Toc157440759)

[3.2 General Preventive Measures 21](#_Toc157440760)

[4. Clinical Features 21](#_Toc157440761)

[4.1 Clinical Manifestation 21](#_Toc157440762)

[4.2 laboratory examination 22](#_Toc157440763)

[4.3 Chest Imaging 22](#_Toc157440764)

[5. Diagnosis 23](#_Toc157440765)

[5.1 Diagnostic Principles 23](#_Toc157440766)

[5.2 Diagnostic Criteria 23](#_Toc157440767)

[6. Clinical classification 23](#_Toc157440768)

[6.1 Mild infection 23](#_Toc157440769)

[6.2 Moderate infection 23](#_Toc157440770)

[6.3 Severe infection 23](#_Toc157440771)

[6.4 Critical 24](#_Toc157440772)

[7. High-risk population for severe/critical conditions 24](#_Toc157440773)

[8. Severe /Critical Early Warning Indicators 24](#_Toc157440774)

[8.1 Adult 24](#_Toc157440775)

[9. Treatment 25](#_Toc157440776)

[9.1 General Treatment 25](#_Toc157440777)

[9.2 Antiviral Treatment 25](#_Toc157440778)

[9.3 Immunotherapy 27](#_Toc157440779)

[9.4 Anticoagulant therapy 27](#_Toc157440780)

[9.5 Prone position therapy 27](#_Toc157440781)

[9.6 Psychological Intervention 27](#_Toc157440782)

[9.7 Supportive Treatment for Severe and Critical Illnesses 28](#_Toc157440783)

[10. Nursing 30](#_Toc157440784)

[11. Infection Prevention and Control within Healthcare Facilities 31](#_Toc157440785)

[12. Discharge Criteria for Hospitalized Patients 31](#_Toc157440786)

1. Pathogenic Characteristics

The novel coronavirus (hereinafter referred to as SARS-CoV-2) belongs to the β genus of coronaviruses, possesses an envelope, and the particles are round or oval in shape with a diameter of 60-140nm. The viral particles contain four structural proteins: spike protein (S), envelope protein (E), membrane protein (M), and nucleocapsid protein (N). The genome of the novel coronavirus is a single-stranded positive-sense RNA, approximately 29.9kb in length. The open reading frames (ORFs) arranged in order are:5′-replicase(ORF1a/ORF1b)-S-ORF3a-ORF3b-E-M-ORF6-ORF7a-ORF7b-ORF8-N-ORF9a-ORF9b-ORF10-3′. Nucleocapsid protein N wraps around the viral RNA, forming the core structure of the virus particle - the nucleocapsid. The nucleocapsid is then enclosed by a double-layered lipid membrane, which is embedded with the S, M, and E proteins of the novel coronavirus. After the novel coronavirus invades the human respiratory tract, it primarily relies on the Receptor Binding Domain (RBD) on its spike protein to identify and bind to the host cell receptor angiotensin-converting enzyme 2 (ACE2), thereby infecting the host cells. During the circulation and transmission of the novel coronavirus among the population, frequent mutations occur in its genes. When different subtypes or descendant branches of the novel coronavirus infect humans simultaneously, recombination can also take place, resulting in recombinant virus strains. Certain mutations or recombinations can affect the biological characteristics of the virus. For example, after specific amino acid mutations on the S protein, the affinity between the novel coronavirus and ACE2 is enhanced, which increases its replication and transmission capabilities within cells. S protein some amino acid mutations will also increase the ability to escape immune response to vaccines and reduce the cross-protection between different sub-branch variants, leading to breakthrough infections and a certain proportion of reinfections. As of the end of 2022, the World Health Organization (WHO) proposed "variants of concern" (VOC) have 5, respectively Alpha (Alpha, B.1.1.7), Beta (Beta, B.1.351), Gamma (Gamma, P.1), Delta (Delta, B.1.617.2) and Omicron (Omicron, B.1.1.529). The Omicron variant emerged among the population in November 2021, and compared to other VOC variants such as Delta, it has significantly enhanced transmissibility and immune evasion capabilities. By early 2022, it rapidly replaced the Delta variant to become the dominant strain globally.

Up to now, five subvariants of Omicron (BA.1, BA.2, BA.3, BA.4, BA.5) have successively evolved into 709 descendant sub-branches, including 72 recombinant branches. With the continuous global spread of the COVID-19 virus, new Omicron sub-branches will continue to emerge. The Omicron variant that has been prevalent globally for several months is primarily BA.5.2. However, since October 2022, subvariants and recombinant strains with stronger immune escape capabilities and transmissibility, such as BF.7, BQ.1, and BQ.1.1 (XBB), have rapidly increased in transmission dominance. They have replaced BA.5.2 as the dominant strain in certain countries and regions.

Evidence from both domestic and international sources indicates that the pathogenicity of the Omicron variant in the lungs has significantly weakened, with clinical manifestations evolving from pneumonia as the primary symptom to upper respiratory tract infections. The diagnostic accuracy of the PCR testing method commonly used within our country remains unaffected; however, the neutralizing effect of some monoclonal antibody drugs that have been developed and marketed has significantly decreased.

COVID-19 is sensitive to ultraviolet light, organic solvents (such as ether, 75% ethanol, peracetic acid, and chloroform), and chlorine-containing disinfectants. 75% ethanol and chlorine-containing disinfectants are commonly used in clinical and laboratory settings for the inactivation of the virus, but chlorhexidine is not effective in inactivating the virus.

1. Epidemiological Characteristics

### Infection Source

The main source of infection is the novel coronavirus carrier, who is contagious even during the incubation period, with the highest infectivity within 3 days after the onset of the disease.

### Transmission Route

i. Transmission through respiratory droplets and close contact is the primary route of transmission.

ii. It can also spread through aerosols in relatively enclosed environments.

iii. Infection can also occur after touching objects contaminated with the virus.

### 2.3 Susceptible Population

The general population is susceptible. After infection or vaccination against the COVID-19 virus, one can acquire a certain level of immunity. The elderly and those with severe underlying diseases have a higher rate of severe illness and mortality after infection compared to the general population, but vaccination can reduce the risk of severe illness and death.

1. Prevention

### COVID-19 Vaccine Inoculation

Vaccination against COVID-19 can reduce the infection and onset of the virus, and is an effective means to lower the incidence of severe cases and mortality. Those who meet the vaccination criteria should all be vaccinated. Individuals eligible for booster immunization should receive it in a timely manner.

### General Preventive Measures

Maintain good personal and environmental hygiene, balance nutrition rest, and avoid excessive fatigue balance nutrition, moderate exercise, adequate rest, and avoid excessive fatigue. Improve health literacy, develop the habit of "one-meter line", frequent hand washing, wearing masks, using serving chopsticks, and other hygiene habits and lifestyles, cover your mouth and nose when sneezing or coughing. Keep indoor ventilation well and do a good job in personal protection.

4. Clinical Features

### 4.1 Clinical Manifestation

The incubation period is mostly 2 to 4 days.

The main symptoms include dry throat, sore throat, cough, and fever. The fever is often mild to moderate, but some cases may present with high fever, which usually does not last more than 3 days. Some patients may also experience muscle soreness, reduced or lost sense of smell and taste, nasal congestion, runny nose, diarrhea, and conjunctivitis. A minority of patients may see their condition progress, with persistent fever and the emergence of pneumonia-related symptoms. Severe cases often develop respiratory distress and/or hypoxemia 5 to 7 days after the onset of illness. In severe cases, the condition can rapidly progress to acute respiratory distress syndrome, septic shock, refractory metabolic acidosis, coagulation dysfunction, and multiple organ failure. A very small number of patients may also exhibit central nervous system involvement.

Most patients have a good prognosis, while those with severe conditions are often seen in the elderly, those with chronic underlying diseases, late pregnancy and perinatal women, obese people, etc.

### 4.2 laboratory examination

#### 4.2.1 General Examination

In the early stages of the disease, the total number of peripheral blood leukocytes may be normal or decreased, with a reduction in lymphocyte count. Some patients may exhibit elevated levels of liver enzymes, lactate dehydrogenase, muscle enzymes, myoglobin, cardiac troponin, and ferritin. A subset of patients may show increased C-reactive protein (CRP) and erythrocyte sedimentation rate (ESR), while procalcitonin (PCT) remains normal. In severe and critical cases, there may be an increase in D-dimer levels, a progressive decrease in peripheral blood lymphocytes, and elevated inflammatory factors.

#### 4.2.2Etiology and Serology Examination

1. Nucleic Acid Testing: Nucleic acid amplification testing methods can be used to detect the nucleic acid of the novel coronavirus in respiratory specimens (nasopharyngeal swabs, oropharyngeal swabs, sputum, tracheal aspirates) or other specimens. Fluorescence quantitative PCR is currently the most commonly used method for detecting the nucleic acid of the novel coronavirus.
2. Antigen testing: Colloidal gold method and immunofluorescence method are used to detect viral antigens in respiratory tract specimens. The detection speed is fast, and its sensitivity is positively correlated with the viral load of the infected person. A positive result of viral antigen testing supports the diagnosis, but a negative result cannot exclude it.
3. Virus culture and isolation: The novel coronavirus can be isolated and cultured from respiratory tract specimens, fecal specimens, etc.
4. Serological testing: Positive for specific IgM antibodies and IgG antibodies of the novel coronavirus, the positive rate within one week of onset is relatively low. The level of IgG antibody during the recovery period is four times or more higher than that during the acute phase, which has retrospective diagnostic significance.

### 4.3 Chest Imaging

Patients with concomitant pneumonia initially present with multiple small patchy opacities and interstitial changes, predominantly in the peripheral lung zones. This may progress to bilateral ground-glass opacities and infiltrative shadows. In severe cases, pulmonary consolidation can occur, while pleural effusion is uncommon.

5. Diagnosis

### 5.1 Diagnostic Principles

According to the comprehensive analysis of epidemiological history, clinical manifestations, and laboratory examinations, a diagnosis is made. A positive nucleic acid test for the novel coronavirus is the primary criterion for confirmation.

### 5.2 Diagnostic Criteria

1. Clinical manifestations related to COVID-19 infection
2. One or more of the following etiological and serological test results
3. Positive result in nucleic acid test for COVID-19 virus;
4. Positive result in antigen test for COVID-19 virus;
5. Positive result in isolation and culture of COVID-19 virus;
6. The level of specific IgG antibody to COVID-19 virus in the convalescence phase is four times or more higher than that in the acute phase.

6. Clinical classification

### 6.1 Mild infection

Respiratory tract infection is the main manifestation, such as dry throat, sore throat, cough, fever, etc.

### 6.2 Moderate infection

Continuous high fever > 3 days or (and) cough, shortness of breath, etc., but respiratory rate (RR) < 30 breaths/min, and oxygen saturation > 93% when breathing air at rest. Imaging studies show characteristic findings of COVID-19 pneumonia infection.

### 6.3 Severe infection

An adult meets any of the following criteria and cannot be explained by reasons other than COVID-19 infection:

1. Shortness of breath occurs, with a respiratory rate (RR) ≥ 30 breaths per minute;
2. At rest, the fingertip oxygen saturation is ≤ 93% when breathing air;
3. The arterial oxygen partial pressure (PaO2) to fraction of inspired oxygen (FiO2) ratio is ≤ 300 mmHg (1 mmHg = 0.133 kPa). In high altitude areas (altitude exceeding 1000 meters), the PaO2/FiO2 should be corrected according to the following formula: PaO2/FiO2 × [760/atmospheric pressure (mmHg)];
4. Clinical symptoms worsen progressively, and lung imaging shows that the lesion has significantly progressed by more than 50% within 24 to 48 hours.

### 6.4 Critical

Those who meet one of the following conditions:

1. Experiencing respiratory failure and requiring mechanical ventilation;
2. Experiencing shock;
3. Combined with other organ failures requiring ICU monitoring and treatment.

7. High-risk population for severe/critical conditions

1. Individuals over the age of 65, especially those who have not been fully vaccinated against COVID-19;
2. Patients with underlying conditions such as cardiovascular and cerebrovascular diseases (including hypertension), chronic pulmonary diseases, diabetes, chronic liver and kidney diseases, tumors, and maintenance hemodialysis patients;
3. Those with immune deficiencies (such as HIV/AIDS patients, long-term users of corticosteroids or other immunosuppressive drugs leading to a reduced immune function);
4. Obese individuals (Body Mass Index ≥ 30);
5. Women in late pregnancy and perinatal period;
6. Heavy smokers.

8. Severe /Critical Early Warning Indicators

### 8.1 Adult

The following changes in indicators should alert to the worsening of the condition:

1. Progressive worsening of hypoxemia or respiratory distress;
2. Deterioration of tissue oxygenation indicators (such as fingertip oxygen saturation, oxygenation index) or progressive increase in lactate;
3. Progressive decrease in peripheral blood lymphocyte count or progressive increase in inflammatory factors such as Interleukin-6 (IL-6), CRP, ferritin, etc.;
4. Significant elevation in coagulation function-related indicators such as D-dimer;
5. Chest imaging shows significant progression of lung lesions.

9. Treatment

### 9.1 General Treatment

1. Isolate and treat according to the requirements of respiratory infectious diseases. Ensure adequate energy and nutrient intake, pay attention to water and electrolyte balance, and maintain internal environmental stability. Physical cooling and antipyretic drugs can be used for high fever. Cough and phlegm can be treated with cough suppressants and expectorants.
2. Vital signs monitoring should be carried out for severe high-risk groups, especially oxygen saturation at rest and after activity. At the same time, monitor related indicators of underlying diseases.
3. Conduct necessary examinations according to the condition, such as routine blood test, urine routine, CRP, biochemical indicators (liver enzymes, myocardial enzymes, renal function, etc.), coagulation function, arterial blood gas analysis, chest imaging, etc.
4. According to the condition, standardized and effective oxygen therapy measures should be given, including nasal catheter, mask oxygenation and high flow nasal oxygen therapy.
5. Antimicrobial drug treatment: Avoid blind or inappropriate use of antimicrobial drugs, especially the combined use of broad-spectrum antimicrobial drugs.
6. Corresponding treatment should be given to those with underlying diseases.

### 9.2 Antiviral Treatment

1. Nirmatrelvir tablets/Ritonavir tablets combination pack (Paxlovid). Suitable for adult patients with mild to moderate symptoms within 5 days of onset and high risk factors for progression to severe illness. Usage: Take Nirmatrelvir 300mg and Ritonavir 100mg simultaneously, once every 12 hours, for a continuous period of 5 days. Before use, the instructions should be read in detail. Do not combine with drugs that are highly dependent on CYP3A for clearance and whose increased plasma concentration can lead to serious and/or life-threatening adverse reactions, such as meperidine and lofexidine. It should only be used during pregnancy if the potential benefits to the mother outweigh the potential risks to the fetus. Not recommended for use during breastfeeding. Patients with moderate renal impairment should take half the dose of Nirmatrelvir, and those with severe liver or renal impairment should not use it.
2. Azvudine tablets. Used for the treatment of adult patients with moderate COVID-19 infection. Usage: Take the whole tablet on an empty stomach, 5mg each time, once a day, with a course of treatment not exceeding 14 days. Before use, read the instructions carefully, and pay attention to issues such as interactions with other drugs and adverse reactions. It is not recommended for use during pregnancy and breastfeeding, and caution should be exercised in patients with moderate to severe liver or kidney damage.
3. Molnupiravir capsules. Suitable for adult patients who have developed mild to moderate symptoms within 5 days and are at high risk of progressing to severe illness. Usage: 800 milligrams, taken orally every 12 hours for 5 consecutive days. Not recommended for use during pregnancy and breastfeeding.
4. Monoclonal antibodies: Ambasivir/Romidepsin monoclonal antibody injection. Combined use for the treatment of adults and adolescents (aged 12-17 years, weighing ≥40kg) with mild to moderate symptoms and at high risk of progressing to severe illness. Usage: The doses of the two drugs are 1000 mg each. Before administration, dilute the two drugs separately with 100 ml of saline, and then infuse them sequentially via intravenous injection, with a drip rate not exceeding 4ml/min. Flush the tube with 100ml of saline between the two drugs. Clinical monitoring of the patient should be carried out during the infusion, and the patient should be observed for at least 1 hour after the infusion is completed.
5. Intravenous COVID-19 human immunoglobulin. Can be used early in the course of the disease for patients with high risk factors for severe illness, high viral load, and rapid progression of the disease. The dosage is 100mg/kg for mild cases, 200mg/kg for moderate cases, and 400mg/kg for severe cases, administered intravenously. Depending on the patient's condition improvement, the treatment can be repeated the next day, with a total number of treatments not exceeding 5 times.
6. Convalescent plasma from recovered patients. Can be used early in the course of the disease for patients with high risk factors for severe illness, high viral load, and rapid progression of the disease. The transfusion dose is 200-500ml (4-5ml/kg), and whether to repeat the transfusion can be decided based on individual patient conditions and viral load.
7. Other anti-COVID-19 drugs approved by the National Medical Products Administration.

### 9.3 Immunotherapy

1. Glucocorticoids: For severe and critical cases with progressive deterioration in oxygenation indices, rapid radiological progression, and an excessively activated state of the body's inflammatory response, glucocorticoids are recommended for short-term use (not exceeding 10 days). Dexamethasone at 5mg/day or methylprednisolone at 40mg/day is suggested. Long-term and high-dose use of glucocorticoids should be avoided to reduce side effects.
2. Interleukin-6 (IL-6) inhibitor: Tocilizumab. It can be tried for severe and critically ill patients with significantly elevated IL-6 levels detected in the laboratory. Dosage: The initial dose is 4-8mg/kg, with a recommended dose of 400mg, diluted in saline to 100ml, and the infusion time should be greater than 1 hour; for those who do not respond well to the first dose, an additional dose (same as before) can be given 12 hours after the initial dose, with a maximum of 2 doses in total, and the maximum single dose should not exceed 800mg. Be aware of allergic reactions, and it is contraindicated in patients with active infections such as tuberculosis.

### 9.4 Anticoagulant therapy

For patients with severe risk factors, rapidly progressing as severe and critical cases, rapidly progressing moderate cases, as well as severe and critical cases, low molecular weight heparin or standard heparin can be administered at therapeutic doses, provided there are no contraindications. In the event of thromboembolic events, treatment should be carried out in accordance with the corresponding guidelines.

### 9.5 Prone position therapy

Patients with high-risk factors for severe illness, moderate, severe, and critical cases with rapid progression should be given standard prone position therapy, recommended for no less than 12 hours per day.

### 9.6 Psychological Intervention

Patients often experience tension and anxiety, and psychological counseling should be strengthened. If necessary, drug treatment should be supplemented.

### 9.7 Supportive Treatment for Severe and Critical Illnesses

- 1. Treatment Principles: On the basis of the above treatments, actively prevent and treat complications, treat underlying diseases, prevent secondary infections, and provide timely organ function support.
  2. Respiratory Support:

1. Nasal Cannula or Mask Oxygenation ：

Patients with severe cases who have a PaO2/FiO2 ratio below 300 mmHg should be given oxygen therapy immediately.After receiving nasal cannula or mask oxygenation, observe closely for a short period (1-2 hours),if respiratory distress and/or hypoxemia do not improve, high-flow nasal cannula oxygen therapy (HFNC) or non-invasive ventilation (NIV) should be used.

1. High-Flow Nasal Cannula Oxygen Therapy or Non-Invasive Ventilation：

Patients with a PaO2/FiO2 ratio below 200 mmHg should be given high-flow nasal cannula oxygen therapy (HFNC)or non-invasive ventilation (NIV). For patients receiving HFNC or NIV, if there are no contraindications,it is recommended to implement prone positioning ventilation simultaneously, that is, awake prone positioning ventilation,and the treatment time in the prone position should be more than 12 hours per day.

Some patients have a high risk of failure in treatment with HFNC or NIV, and it is necessary to closely monitor their symptoms and signs. If there is no improvement in the condition after a short period (1-2 hours) of treatment, especially after receiving prone position therapy, hypoxemia remains unimproved, or there are excessive respiratory rate, tidal volume, or strong inspiratory effort, it often indicates that HFNC or NIV treatment is not effective, and invasive mechanical ventilation should be carried out in a timely manner.

1. Invasive Mechanical Ventilation：

Under normal circumstances, when the PaO2/FiO2 ratio falls below 150mmHg, especially in patients with significantly increased inspiratory effort, consideration should be given to tracheal intubation and the implementation of invasive mechanical ventilation. However, given that some severe and critical cases have atypical clinical manifestations of hypoxemia, the decision for tracheal intubation and invasive mechanical ventilation should not solely rely on whether the PaO2/FiO2 ratio meets the standard. Instead, it should be based on a real-time assessment that combines the patient's clinical presentation and organ function status. It is worth noting that delayed tracheal intubation could lead to even greater harm.

Early appropriate invasive mechanical ventilation treatment is an important therapeutic approach for critical cases, and a lung-protective mechanical ventilation strategy should be implemented. For patients with moderate to severe acute respiratory distress syndrome, or when the FiO2 of invasive mechanical ventilation exceeds 50%, lung recruitment therapy can be applied, and whether to repeatedly implement lung recruitment maneuvers should be determined based on the responsiveness to lung recruitment. It should be noted that some patients with novel coronavirus infections have poor lung recruitability, and excessive PEEP should be avoided to prevent barotrauma.

1. Airway Management

Strengthen airway humidification, it is recommended to use active heated humidifiers, and if possible, use loop heating guide wires to ensure the effect of humidification; it is recommended to use closed aspiration, and when necessary, tracheoscopic aspiration; actively carry out airway clearance treatment, such as vibration expectoration, high-frequency chest wall oscillation, postural drainage, etc.; under the condition of oxygenation and hemodynamic stability, passive and active activities should be carried out as soon as possible to promote sputum drainage and lung rehabilitation.

1. Extracorporeal Membrane Oxygenation (ECMO)

ECMO Initiation Timing: Under optimal mechanical ventilation conditions (FiO2≥80%, tidal volume of 6ml/kg ideal body weight, PEEP≥5cmH2O, and no contraindications), and when protective ventilation and prone position ventilation are ineffective, and one of the following criteria is met, ECMO should be considered for early assessment and implementation.

① PaO2/FiO2＜50mmHg for more than 3 hours;

② PaO2/FiO2＜80mmHg for more than 6 hours;

③ Arterial blood pH＜7.25 and PaCO2＞60mmHg for more than 6 hours, with a respiratory rate＞35 breaths/min;

④ When the respiratory rate is＞35 breaths/min, arterial blood pH＜7.2 and plateau pressure＞30cmH2O.

Critical cases that meet the indications for ECMO and have no contraindications should initiate ECMO treatment as soon as possible to avoid delaying the timing and leading to poor patient prognosis. ECMO mode selection. When only respiratory support is needed, veno-venous ECMO (VV-ECMO) is the most commonly used method; when both respiratory and circulatory support are needed, veno-arterial ECMO (VA-ECMO) is chosen; when VA-ECMO causes hypoxia in the head and arm area, veno-arterial-venous ECMO (VAV-ECMO) can be used. After implementing ECMO, strictly implement a protective lung ventilation strategy. Recommended initial settings: Tidal volume＜4～6ml/kg ideal body weight, plateau pressure≤25cmH2O, driving pressure＜15cmH2O, PEEP5～15cmH2O, respiratory rate 4～10 breaths/min, FiO2＜50%. For patients who have difficulty maintaining oxygenation function or have strong inspiratory efforts, obvious consolidation in the dependent areas of both lungs, or need airway secretion drainage, prone position ventilation should be actively implemented.

- 1. Circulatory Support：Critical cases may be complicated by shock. On the basis of adequate fluid resuscitation, vasoactive drugs should be used appropriately, closely monitoring changes in patient's blood pressure, heart rate, and urine output, as well as lactate and base excess. Hemodynamic monitoring should be performed when necessary.
  2. Acute Kidney Injury and Renal Replacement Therapy：Severe cases may be complicated by acute kidney injury, and it is essential to actively identify the causes, such as hypoperfusion and medications. While actively correcting the causes, attention should be paid to maintaining water, electrolyte, and acid-base balance. Indications for continuous renal replacement therapy (CRRT) include:

① hyperkalemia;

② severe acidosis;

③ pulmonary edema or excessive fluid overload unresponsive to diuretics.

10. Nursing

Based on the patient's condition, clarify the focus of nursing and ensure proper basic care. For severe cases, closely monitor vital signs and consciousness state, with a particular emphasis on monitoring blood oxygen saturation. In critical cases, implement continuous 24-hour ECG monitoring, measuring the patient's heart rate, respiratory rate, blood pressure, and blood oxygen saturation (SpO2) hourly, and record body temperature every 4 hours. Use intravenous access appropriately and correctly, ensuring that all types of catheters are unobstructed and securely fastened. Bedridden patients should change positions regularly to prevent pressure injuries. Follow nursing standards for non-invasive mechanical ventilation, invasive mechanical ventilation, artificial airways, prone position ventilation, sedation and analgesia, and ECMO therapy. Pay special attention to oral care for patients and management of fluid intake and output, preventing aspiration in patients on invasive mechanical ventilation. Assess the psychological status of conscious patients in a timely manner and provide proper psychological care.

11. Infection Prevention and Control within Healthcare Facilities

1. Implement the pre-examination and triage system for outpatient and emergency departments, and properly divert patients. Provide guidance on hand hygiene, respiratory hygiene, and cough etiquette; patients with respiratory symptoms and their companions should wear surgical masks or medical protective masks.
2. Strengthen ventilation in wards, and ensure the cleanliness and disinfection of surfaces in areas such as examination rooms, wards, offices, and duty rooms.
3. Medical personnel should follow standard precautionary principles, taking appropriate personal protection based on exposure risk. Wear surgical masks or medical protective masks during work, and strictly adhere to hand hygiene practices.
4. Handle medical waste according to requirements, and perform terminal disinfection after patients are transferred out or discharged from the hospital.

12. Discharge Criteria for Hospitalized Patients

The patient's condition has significantly improved, vital temperature has been normal for over body temperature has been normal for over 24 hours, and lung imaging shows significant improvement in acute exudative lesions. Oral medication treatment can be considered, and there are no complications requiring further intervention. Discharge may be considered under these circumstances.

**APPENDIX II**

**Informed consent (Chinese version)**

**研究名称：EGCG雾化吸入治疗新冠病毒肺炎的I/II期临床试验**

**申办单位：山东省肿瘤医院**

**患者姓名缩写：**

**患者研究编号：**

**患者联系电话：**

**患者联系地址：**

**EGCG雾化吸入治疗新冠病毒感染/新冠病毒肺炎知情同意书**

**2023-1-3（第一版）**

**疾病分类：**

**新冠病毒感染：**有明确流行病学史，出现发热和/或呼吸道症状等临床表现，且新冠病毒核酸/抗原阳性。

**新冠病毒肺炎：**诊断为新冠病毒感染，且肺部出现符合新冠病毒感染所致肺炎的新发影像学异常。

**病情分级**

综合考虑氧合及呼吸困难情况、重症危险因素（免疫抑制、基础疾病、高龄）、肺炎广泛程度和进展速度，可将感染者分为轻症、重症和危重症。

**合并肺炎的轻症患者的抗病毒治疗**

部分轻症患者也可有肺炎表现，若无其他重症表现或危险因素，肺炎表现并不一定提示不良预后。X线对筛查肺炎具有良好效果且危害更小，性价比更高。出现肺炎的影像表现但无其他危重症因素患者并不构成住院指征。

对于无危险因素的人群（如既往健康的年轻人群）建议密切监测指氧饱和度，但不建议常规进行CT检查，也不建议常规加用激素。若监测指氧饱和度等指标发现患者转为重症，则按重症处理。

**激素**

无需住院的非重症患者不建议常规使用激素，由主诊医师根据患者的具体情况决定；氧合下降或吸氧需求上升的部分门诊患者可使用地塞米松或其他种类激素；使用激素时需密切随访病情变化和不良反应；感染诱发哮喘急性加重或慢性阻塞性肺疾病急性加重的患者可按相应指南使用激素。

目前临床可及的用于住院患者的药物主要有激素，抗病毒治疗药物如阿兹夫定、奈玛特韦/洛匹那韦、莫诺拉韦，免疫调节治疗药物如托珠单抗、巴瑞替尼、托法替布，暂不可及的药物为瑞德西韦（Remdesivir）。

其他治疗方法包括但不限于：**氧疗和机械通气的治疗；IL-6拮抗剂；JAK抑制剂；抗细菌药物；抗凝药物；恢复期血浆治疗等。**

动物及临床试验表明，表没食子儿茶素没食子酸酯(EGCG)有很高的安全性，其中研究报道小鼠口服半数致死量 (LD50)为2170mg/kg，在慢性淋巴细胞白血病患者中口服应用时每日剂量达4000mg未见到明显的不良反应；EGCG在2010年10月29日被原国家卫生部批准为新资源食品，建议口服剂量≤300mg/日，在本实验中患者口服EGCG的剂量大致为60mg/日，远小于卫生部推荐剂量。

**您的权利**

您参与研究完全是自愿的，您可以在研究任何阶段退出而无需理由，绝不影响您和医务人员的关系及今后的治疗。您不是必须参加本研究。

最后，感谢您对本研究的大力支持，和对该疾病诊疗研究的探索作出的贡献！

**同意声明：**

我已了解了本研究的目的，过程，可能获得的益处和可能发生的不良反应，自愿参加此项研究，并尽量遵从研究流程。

受试者签名： 日期： 年 月 日

联系电话：

研究者签名： 日期： 年 月 日

联系电话（手机）：
